# Draft Genome Sequence of the Probiotic Strain Lactobacillus acidophilus ATCC 4356

**DOI:** 10.1128/genomeA.01421-14

**Published:** 2015-01-15

**Authors:** Maria Mercedes Palomino, Mariana C. Allievi, Joaquina Fina Martin, Pablo M. Waehner, Mariano Prado Acosta, Carmen Sanchez Rivas, Sandra M. Ruzal

**Affiliations:** Universidad de Buenos Aires, Facultad de Ciencias Exactas y Naturales, Departamento de Química Biológica, IQUIBICEN-CONICET, Buenos Aires, Argentina

## Abstract

We present the 1,956,699-bp draft genome sequence of Lactobacillus acidophilus strain ATCC 4356. Comparative genomic analysis revealed 99.96% similarity with L. acidophilus NCFM NC_006814.3 and 99.97% with La-14 NC_021181.2 genomes.

## GENOME ANNOUNCEMENT

The original Lactobacillus acidophilus strain ATCC 4356 was isolated in 1900 (1, 2) from human infant feces. This strain is available from the American Type Culture Collection (http://www.atcc.org/Products/All/4356.aspx). L. acidophilus ATCC 4356 is an important inhabitant of the gastrointestinal tract with reported probiotic properties (3–5). The characterization of its probiotic attributes is a relevant goal. In our lab, we are interested in the cell envelope structure (6, 7), and we have found candidate probiotic functions for its surface-layer protein (8–10).

The strain did not contain any plasmid but did contain a unique genome. The genome sequence was obtained using a whole-genome shotgun strategy with a 454 GS Titanium pyrosequencer at the Instituto de Agrobiotecnología Rosario (INDEAR), Argentina. Assembly was done using 454 Newbler version 2.6, with a genome coverage of 20×. This assembly generated 20 scaffolds using Mauve version 2.3 and the L. acidophilus NCFM genome as a template. The draft genome is 1,956,699 bp in length, and the G+C content is 34.6%. Genomic annotations were assigned automatically by the NCBI Prokaryotic Genomes Automatic Annotation Pipeline (PGAAP) with some minor additions from Rapid Annotations using Subsystems Technology (RAST) (11, 12). The annotation predicted 1,977 coding sequences (CDSs) and 62 structural RNAs (59 tRNAs). A total of 506 CDSs (25.6%) were classified as hypothetical proteins. According to RASTtk, the annotation identified 903 CDSs (46%) into RAST subsystems. The genome also contains 1 CRISPR array.

Comparative genomic analysis revealed 99.96% similarity with L. acidophilus NCFM NC_006814.3 (13) and 99.97% similarity with La-14 NC_021181.2 (14); the genomes also shared high synteny. BLASTn analysis of the 29 sequences of this strain deposited in GenBank shows that 22 out of 29 presented between 99 and 100% identity. Differences would rely on the methods employed since higher identity was observed for the more recent nucleotide sequences.

A differential feature never annotated before in Lactobacillus acidophilus strains was the presence of a coding sequence for chitinase activity (EC 2.7.1.69, NH13_08655). In fact, several chitin and *N*-acetylglucosamine utilization functions were predicted by RASTtk, particularly the PTS system for *N*-acetylglucosamine-specific IIABC components (NH13_02050), as well as the *N*-acetyl-d-glucosamine ABC transport system, permease protein (NH13_08155). Mechanisms of the enzymes degrading *N*-acetylglucosamine (15) are predicted: NagB, GlcN6P deaminase (NH13_09645) and NagA, GlcNAc6P deacetylase (NH13_00815). A transcriptional regulator of *N*-acetylglucosamine utilization (NH13_02010) from the GntR family was also found. We confirm their functionality: cells were able to grow in chitin as the sole carbon source and in plates containing chitin and stained with calcofluor, and a degradation halo was observed. Moreover, the hydrolytic activity was found in the cell wall fractions. The presence of chitinase might constitute a system for scavenging mucosa-derived carbohydrates, as well as an adherence factor, since GlcNAc is found in many human glycoproteins at mucosal surfaces and probably represents an adaptation for development at the intestinal mucosal niche.

### Nucleotide sequence accession numbers.

This whole-genome shotgun project has been deposited at DDBJ/EMBL/GenBank under the accession number JRUT00000000. The version described in this paper is version JRUT01000000.
